# Protein Arginine Methyltransferase 5 Promotes Esophageal Squamous Cell Carcinoma Proliferation and Metastasis via LKB1/AMPK/mTOR Signaling Pathway

**DOI:** 10.3389/fbioe.2021.645375

**Published:** 2021-05-28

**Authors:** Yu-ru Chen, Hua-ni Li, Lian-jun Zhang, Chong Zhang, Jin-guang He

**Affiliations:** ^1^Department of Oncology, Heze Municipal Hospital, Heze, China; ^2^Department of Critical Care Medicine, Heze Municipal Hospital, Heze, China; ^3^Magnetic Resonance Room, Heze Municipal Hospital, Heze, China

**Keywords:** protein arginine methyltransferase 5, esophageal squamous cell carcinoma, proliferation, metastasis, LKB1/AMPK/mTOR pathway signaling

## Abstract

**Background:** Esophageal squamous cell carcinoma (ESCC) is the eighth most common cancer in the world. Protein arginine methyltransferase 5 (PRMT5), an enzyme that catalyzes symmetric and asymmetric methylation on arginine residues of histone and non-histone proteins, is overexpressed in many cancers. However, whether or not PRMT5 participates in the regulation of ESCC remains largely unclear.

**Methods:** PRMT5 mRNA and protein expression in ESCC tissues and cell lines were examined by RT-PCR, western blotting, and immunohistochemistry assays. Cell proliferation was examined by RT-PCR, western blotting, immunohistochemistry assays, MTT, and EdU assays. Cell apoptosis and cell cycle were examined by RT-PCR, western blotting, immunohistochemistry assays, and flow cytometry. Cell migration and invasion were examined by RT-PCR, western blotting, immunohistochemistry assays, and wound-healing and transwell assays. Tumor volume, tumors, and mouse weight were measured in different groups. Lung tissues with metastatic foci, the number of nodules, and lung/total weight were measured in different groups.

**Results:** In the present study, the PRMT5 expression level was dramatically upregulated in ESCC clinical tissues as well as ESCC cell lines (ECA109 and KYSE150). Furthermore, knocking down PRMT5 obviously suppressed cell migration, invasion, proliferation, and cell arrest in G1 phase and promoted cell apoptosis in ESCC cells. Meanwhile, downregulating PRMT5 also increased the expression levels of Bax, caspase-3, and caspase-9, while expression levels of Bax-2, MMP-2, MMP-9, and p21 were decreased, which are members of the cyclin-dependent kinase family. Furthermore, knocking down PRMT5 could increase the expression of LKB1 and the phosphorylation (p)-AMPK expression and decrease the p-mTOR level. Additionally, overexpression of LKB1 could reveal anti-tumor effects in ESCC cell lines by inhibiting ESCC cell, migration, invasion, and proliferation and accelerating cell apoptosis. Besides, upregulating LKB1 expression could increase the levels of Bax, caspase-3, and caspase-9 and weaken the levels of Bax-2, MMP-2, and MMP-9. Moreover, knocking down PRMT5 could weaken the tumor growth and lung metastasis *in vivo* with upregulating the LKB1 expression and the p-AMPK level and downregulating the p-mTOR expression.

**Conclusion:** PRMT5 may act as a tumor-inducing agent in ESCC by modulating LKB1/AMPK/mTOR pathway signaling.

## Background

The incidence of esophageal squamous cell carcinoma (ESCC) ranks eighth in all cancers worldwide, and the mortality rate ranks sixth (Tang et al., 2019). Due to tumor recurrence and postoperative lymph node metastasis, it is a cancer that develops rapidly and has a poor prognosis, which is common in advanced diseases (Qin et al., 2019). Thus, it is urgent to raise awareness of the underlying mechanisms of ESCC tumorigenesis and development and then to pursue new therapeutic targets.

Protein arginine methyltransferase (PRMT) is a post-translational modification that is closely related to gene transcription and signal transduction (Blanc and Richard, 2017). PRMT 5 (PRMT5) is an enzyme that catalyzes the methylation of arginines on histones and non-histones (Vinet et al., 2019). PRMT5, targeting histones H3 and H4, can significantly alter gene expression programs to promote cell transformation (Pal et al., 2007; Chung et al., 2013). PRMT5 is overexpressed in many cancers, such as melanoma, lung, and gastric cancers; and overexpression of PRMT5 is usually associated with poor prognosis (Stopa et al., 2015). It was found that PRMT5 could promote hepatocellular carcinoma proliferation by downregulating BTG2 expression via the ERK pathway (Jiang et al., 2018). In addition, PRMT5 is heterogeneously expressed in triple-negative breast cancer (TNBC); and inhibition of PRMT5 triggers apoptosis and regulates cell cycle progression (Vinet et al., 2019). Besides, PRMT5 regulates pro-survival genes expressions by upregulating WNT/β-CATENIN and AKT/GSK3β signaling pathways; and inhibiting PRMT5 can induce lymphoma cell death (Chung et al., 2019). PRMT5 could be repressed by *N*-alpha-acetyltransferase 40 (NAA40), which results in downregulating key oncogene expressions, upregulating tumor suppressor genes levels, and finally leading to inhibiting colorectal cancer cell growth (Demetriadou et al., 2019). Additionally, PRMT5 could promote human lung cancer cell proliferation through direct interaction with AKT and regulation of AKT activity (Zhang S. et al., 2019). Meanwhile, the epigenetic regulation of PRMT5 through FOXP1 is a key regulator of breast cancer stem cell survival (Chiang and Davies, 2018). It was found that arginine methyltransferase inhibitor 1 inhibits gastric cancer by downregulating eIF4E and targeting type II PRMT5 (Zhang et al., 2017). Moreover, PRMT5 works together to promote cell invasion and metastasis of ESCC, which are targeted by miR-106b-25 clusters (Wang M. et al., 2018). Meanwhile, high PRMT5 expression is usually accompanied by a higher nuclear liver kinase B1 (LKB1). In addition, a number of evidences indicate that PRMT5 and LKB1 interact directly in mammary epithelial cells (Lattouf et al., 2019). However, whether or not PRMT5 participates in the regulation of ESCC remains largely unclear.

Liver kinase B1 (also known as STK11) is a tumor suppressor gene that is inactivated in many malignant tumors (Zhang et al., 2021). LKB1 has recently gained significant attention in delineating the complex mechanisms underlying malignant processes such as cell motility, liposome function, protein translation, and various signal transduction pathways (Ciccarese et al., 2019; Bi et al., 2021). However, it is still unclear which of them is related to ESCC tumorigenesis.

In recent years, the significance of genes and the thereof dependent signaling pathways for cancer has been reported (Kefas et al., 2008; Kovalchuk et al., 2008; Esquela-Kerscher, 2011). Alexander et al. indicated that miR-451 modulated the signaling of the LKB1/AMPK (AMP activated kinase)/mammalian target of rapamycin (mTOR) pathway (Alexander and Walker, 2011; Li et al., 2019). Many researchers have found that this intracellular pathway is involved in cell function (Godlewski et al., 2010). The current study aimed to investigate the role and mechanism of PRMT5 via LKB1/AMPK/mTOR signaling pathway on ESCC.

## Materials and Methods

### Reagents

RPMI-1640 medium and fetal bovine serum (FBS) were obtained from Thermo Fisher Scientific (Waltham, MA, United States). Matrigel was purchased from BD Biosciences (San Jose, CA, United States). Antibodies against Bax, Bax-2, caspase-3, caspase-9, p21 MMP-2, MMP-9, PRMT5, LKB1, AMPK, phosphorylation (p)-AMPK, mTOR, p-mTOR, and GAPDH and secondary antibody were purchased from Abcam (Cambridge, MA, United States).

### Tissue Specimens

Esophageal squamous cell carcinoma tissue samples and paired adjacent normal tissue samples were collected from the same 40 patients who underwent surgery at the Heze Municipal Hospital between June 2017 and December 2018. The tissues were determined by immunohistochemical analysis at the Heze Municipal Hospital. All patients have given written informed consents, and the study protocol was approved by Heze Municipal Hospital. The clinic information of patients is shown in Table 1; and we tested the expression level of PRMT5 in 40 patient samples and selected the median as the classification standard, low expression for those lower than the median, and high expression for those higher than the median.

**TABLE 1:** Association of PRMT5 expression with clinicopathological features of patients with ESCC.

**Characteristics**	**Number**	**Low (*n* = 20)**	**High (*n* = 20)**	***P* value**
Sex				0.691
Male	23	6	17	
Female	17	5	12	
Age				0.045
≤60	27	14	13	
>60	13	6	7	
Tumor size, cm				0.0069
≤4	32	16	16	
>4	8	3	5	
Pathological staging				0.0474
I + II	27	13	14	
III + IV	13	5	8	
Metastasis				0.0267
Yes	26	12	14	
No	14	7	7	

### Cell Culture

The ESCC cell lines ECA109 and KYSE150 and the human normal esophageal epithelial cell line Het-1A were purchased from the Shanghai Institute of Cell Biology (Chinese Academy of Sciences, Shanghai, China). All cells were cultured in RPMI-1640 medium supplemented with 10% FBS at 37°C. Cells were tested for mycoplasma (MycoAlert Mycoplasma Detection Kit, Lonza) and certified mycoplasma free.

### RNA Isolation and Quantitative Real-Time PCR

TRIzol reagent (Invitrogen; Thermo Fisher Scientific, Inc.) was used to extract total RNA from tissue samples and cells. Equal amounts of RNA were transcribed into cDNA using the cDNA First Strand Synthesis kit (Beyotime Institute of Biotechnology, Haimen, China). Total cDNA was used as a starting material for real-time PCR on a StepOne real-time PCR system (Life Technologies Corp.) using a FastStart Universal SYBR Green Master (Roche Applied Science, Mannheim, Germany). The 2^–Δ^ ^Δ^ ^*CT*^ comparative method was used to normalize the expression levels of each target gene to corresponding GAPDH threshold cycle (CT) value. The primers are as follows: PRMT5: forward 5′-TGTAGGGAGAAGGACCGTGA-3′; PRMT5: reverse 5′-ATGGCTGAAGGTGAAACAGG-3′; LKB1: forward 5′-AGGGATGCTTGAGTACGAACC-3′; LKB1: reverse 5′-GTCCTCCAAGTACGGCACC-3′; GAPDH: forward 5′-ACGGATTTGGTCGTATTGGGCG-3′; GAPDH: reverse 5′-CTCCTGGAAGATGGTGATGG-3′.

### Western Blotting

Experimental method was as described above (Chung et al., 2019). Briefly, radioimmunoprecipitation assay (RIPA) lysis buffer was used to lyse the total protein from tissues or cells. Samples were fractionated on a 10% sodium dodecyl sulfate (SDS)–polyacrylamide gels, transferred to polyvinylidene difluoride membranes, and blocked in 5% skim milk in TBST. Subsequently, the membrane was incubated with 1:1,000 dilutions (v/v) primary antibody overnight at 4°C. After the membrane was washed with TBST, it was incubated with a second antibody. Detection was performed using a ChemiDoc Imaging System (Bio-Rad Laboratories, Inc., Hercules, CA, United States). The relative protein expression was analyzed using GAPDH as a control.

### Immunohistochemistry

Experimental method was as described above (Chung et al., 2019). Briefly, lung samples were fixed and cut into 4-μm-thick sections, dried, deparaffinized, and dehydrated in a graded ethanol series and finally incubated with H_2_O_2_. Following blocking with bovine serum, sections were incubated overnight with monoclonal antibodies (1:1,000 dilutions) of PRMT5, LKB1, and Ki-67. The secondary antibody (1:500 dilutions) was used to incubate the sections, and then the sections were incubated with horseradish peroxidase-conjugated streptavidin. A 3,3′-diaminobenzidine (DAB) substrate kit was used, and slides were counterstained with hematoxylin. The laser scanning confocal microscope (Olympus Corporation, Tokyo, Japan) was used to visualize fluorescence.

### Immunofluorescence

Transfected cells (2 × 10^5^) were cultured for 24 h at 37°C on a Petri dish and fixed in 4% formaldehyde. After phosphate-buffered saline (PBS) washes, cells were penetrated and blocked with 5% bovine serum albumin (BSA) for 1.5 h. Then, the cells were incubated with the primary antibody (1:1,000) overnight at 4°C and then incubated with the secondary antibody (1:500) for 30 min. DAPI (1:1,000) was used to stain the nuclei. The laser scanning confocal microscope was used to visualize fluorescence.

### Cell Transfection

PRMT5 siRNA duplexes were obtained from Dharmacon RNA Technologies (Chicago, IL, United States). PcDNA-LKB1 and pcDNA3.1 NC were purchased from GenePharma Company (Shanghai, China). ECA109 and KYSE150 were seeded in six-well plates at a density of 2 × 10^5^ cells and transfected with siRNAs or pcDNA-LKB1 for 24 h at 37°C using Lipofectamine 2000 (Life Technologies). Transfected cells were cultured for 48 h and used for further experiments. The sequence of siRNA PRMT5: 5′-ACAAAGUCCGGAAGUUGUGCC-3′; 3′-CACAACUUCCGGACUUUGUGU-5′.

### Cell Proliferation Assay

For Cell Counting Kit-8 (CCK-8) assay, transfected cells (2 × 10^5^) were seeded in 96-well plates and cultured for 0, 24, 48, and 72 h at 37°C. After treatment with CCK-8 (KeyGEN, Jiangsu, China), optical density (OD) was measured at 450 nm at each point using a microplate reader (Thermo Fisher Scientific, Waltham, MA, United States).

### 5-Ethynyl-2′-Deoxyuridine Staining

5-Ethynyl-2′-deoxyuridine (EdU) incorporation proliferation assay was carried out to evaluate the proliferation of ECA109 and KYSE150 cells. EdU staining was conducted using Cell-Light EdU Imaging detecting kit (Ruibo Biotech, Guangzhou, China) according to the manufacturer’s protocol. The ratio of EdU-positive nuclei to total nuclei was calculated as the proliferation rate of cells in five random high-power fields per well, and the cells were visualized using a fluorescence microscope.

### Apoptosis and Cell Cycle Analysis

The effect of PRMT5 on the apoptosis of ECA109 and KYSE150 cells was evaluated by flow cytometry using the Annexin V Apoptosis Detection kit I (BD Biosciences, San Jose, CA, United States), and the effect of PRMT5 on cell cycle was detected with Cell Cycle Kit. After transfection for 24 h at 37°C, ECA-109 and KYSE150 cells (2 × 10^5^) were digested and washed twice in PBS. Then they were incubated with fluorescein isothiocyanate (FITC) annexin V and propidium iodide (PI) in the dark. The percentage of apoptotic cells and cell cycle was evaluated by FACS Calibur (BD Biosciences, United States).

### Wound-Healing Assay

Transfected ECA109 and KYSE150 cells (2 × 10^5^) were seeded in a six-well plate at 37°C until the fusion reached over 90% and a 200-μl pipette was used to create a linear wound. Then cells were cultured in serum-free Dulbecco’s modified Eagle’s medium (DMEM) (Gibco; Thermo Fisher Scientific, Inc.) at 37°C. After 24 and 48 h, an inverted microscope was used to observe cell migration and to count the number of cells that migrated. Cell migration was assessed by calculating scratch width using the following formula: the relative scratch width = (number of cells at T48 - number of cells at T0)/number of cells at T0 × 100%, where T0 was 0 h and T48 was 48 h.

### Transwell Assay

Experimental method was as described above (Wang et al., 2020). (1) Migration assay: transfected ECA109 and KYSE150 cells (2 × 10^5^) were seeded to the top chamber at a density of 6 × 10^3^ cells, and medium supplemented with 20% FBS and added to the bottom chamber. Twenty-four hours after incubation at 37°C, cells were fixed with 4% formaldehyde and stained with 0.1% crystal violet solution (Servicebio, Wuhan, China). After PBS washing, a microscope was used to count the number of cells. (2) Invasion assays: filters were precoated with Matrigel (BD Biosciences, San Jose, CA, United States), and then transfected cells were plated into the upper chamber. Other operations are the same as above.

### Xenograft Model

A total of 100 μl 2 × 10^6^ KYSE150 cells were dissolved in the Matrigel (BD Biosciences, San Jose, CA, United States) and stably expressed blank, control-siRNA, or PRMT5 siRNA. Then, the cells were injected into the axilla of the male Balb/c nude mice (total of 18; *n* = 6, 6 weeks old, 18–22 g), which were obtained from Beijing Vitalriver Experimental Animal Technology Co., Ltd. The animals were housed under room temperature (18–25°C) and a 12-h light/dark cycle with food and water *ad libitum*. Animal welfare and experimental procedures complied with national guide and was approved by the Heze Municipal Hospital. Animal health and behavior were monitored every day, and the weight of mice was measured every 4 days. When the mice show obvious decrease in activity, abnormal diet, emaciation, and continuous weight loss, they should be immediately anesthetized and sacrificed according to humane endpoints. The experimental mice were routinely monitored and sacrificed on day 32 by cervical dislocation after an intraperitoneal injection of sodium pentobarbital (100 mg/kg). The tumors were removed, and the length and width of the tumors were manually monitored using a Vernier caliper. For lung metastasis model, mice (total of 18; *n* = 6, 6 weeks old, 18–22 g) were injected 100 μl 2 × 10^5^ KYSE150 cells into their tail vein. After mice were sacrificed on day 32, the lung tissues were removed and weighed.

### H&E Staining

Tumor tissues were fixed in 4% paraformaldehyde, embedded in paraffin, and serially sectioned. This section was stained with hematoxylin/eosin (H&E).

### TUNEL Staining

With the use of a TUNEL assay kit (Roche, United States) to detect the apoptosis of tumor sections, briefly, sections were deparaffinized, then permeabilized with proteinase K, treated with 3% H_2_O_2_, and incubated with terminal deoxynucleotidyl transferase (TdT) enzyme. Then, sections were incubated with antidigoxigenin–peroxidase conjugate and using DAB to evaluate activity. The sections were examined under an optical microscope.

### Statistical Analysis

Statistical analysis was performed using GraphPad Prism 5 software. Data are shown as mean and SD. Chi-square test was performed for continuous or discrete data analysis, and the Student paired *t*-test was performed to evaluate significant differences between two independent groups of samples. One-way analysis of variance (ANOVA) followed by Tukey’s *post hoc* test was applied to compare differences among multiple groups. *P* < 0.05 was considered statistically significant.

## Results

### PRMT5 Was Upregulated in Esophageal Squamous Cell Carcinoma Tissues and Cell Lines

**FIGURE 1:**
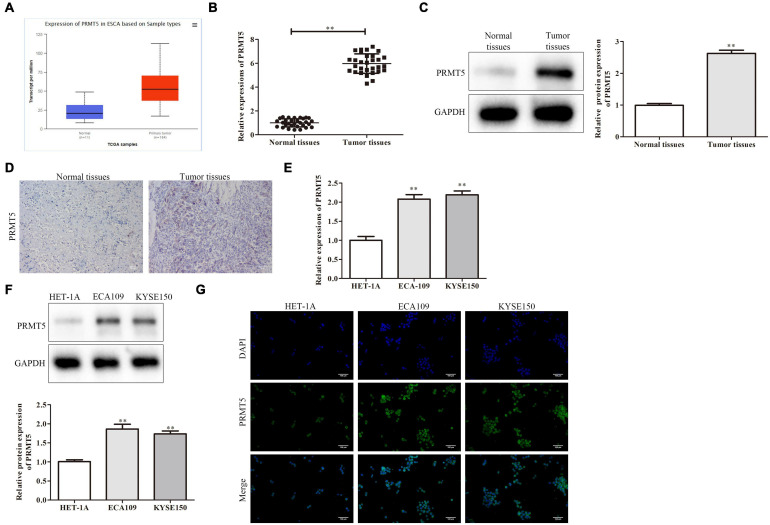
PRMT5 was upregulated in ESCC tissues and cell lines. **(A)** The expression of PRMT5 was analyzed by using GEPIA database. **(B)** PRMT5 mRNA and protein expression in ESCC tissues and normal tissues were examined by RT-PCR assay. **(C)** PRMT5 mRNA and protein expression in ESCC tissues and normal tissues were examined by western blotting assay. **(D)** PRMT5 mRNA and protein expression in ESCC tissues and normal tissues were examined by immunohistochemistry assay. **(E)** PRMT5 expression in ESCC cell lines and human normal esophageal epithelial cell line was examined by RT-PCR assay. **(F)** PRMT5 expression in ESCC cell lines and human normal esophageal epithelial cell line was examined by western blotting assays. **(G)** PRMT5 expression in ESCC cell lines and human normal esophageal epithelial cell line was examined by immunofluorescence assays. Bars indicate the mean ± SEM, **P < 0.01 vs. normal or HET-1A group. ESCC, esophageal squamous cell carcinoma; PRMT5, protein arginine methyltransferase 5; GEPIA, Gene Expression Profiling Interactive Analysis.

Initially, we found PRMT5 is an oncogenic lncRNA in ESCC based on The Cancer Genome Atlas (TCGA) databases. The result also revealed that PRMT5 was significantly upregulated in ESCC tissues compared with normal tissues by using the Gene Expression Profiling Interactive Analysis (GEPIA) database^1^ (Figure 1A). Moreover, RT-PCR analysis showed the PRMT5 expression was remarkably stronger in ESCC tissues than the control tissues, as well as western blotting and immunohistochemistry (IHC) assays (Figures 1B–D). The correlations between the expressions of PRMT5 of the clinic pathological characteristics of 40 patients with ESCC were analyzed. We tested the expression level of PRMT5 in 40 patient samples and selected the median as the classification standard, low expression for those lower than the median, and high expression for those higher than the median. As shown in Table 1, there were significant associations between PRMT5 levels with tumor size, pathological staging, and metastasis. However, there was no association with age and gender. Meanwhile, compared with the HET-1A cell line, a human esophageal epithelial cell line, the PRMT5 expression, was higher in the ESCC cell lines (ECA109 and KYSE150) (Figures 1E,F). Western blotting showed the same result that PRMT5 was upregulated in ESCC tissues and cell lines (ECA109 and KYSE150). In addition, the result of IHC also revealed that the PRMT5 expression was remarkably stronger in ESCC tissues than the control tissues, while the result of immunofluorescence confirmed that PRMT5 was overexpressed in the ESCC cell lines (Figure 1G).

### PRMT5 Promoted Esophageal Squamous Cell Carcinoma Cell Proliferation

**FIGURE 2:**
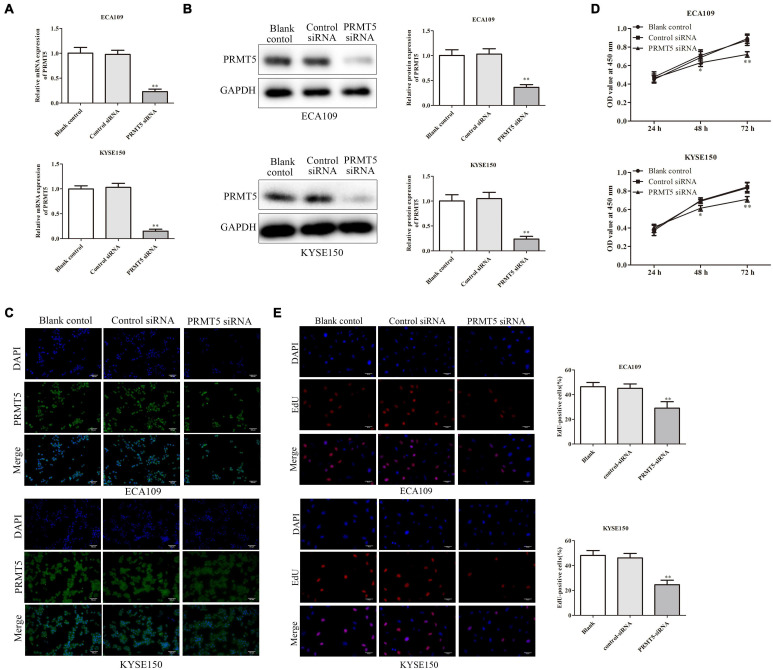
PRMT5 promoted ESCC cell proliferation. **(A–C)** RT-PCR assay indicated that ECA109 and KYSE150 cells transfected with siRNA PRMT5 significantly downregulated the PRMT5 expression compared with the controls. **(B)** Western blotting assay indicated that ECA109 and KYSE150 cells transfected with siRNA PRMT5 significantly downregulated the PRMT5 expression compared with the controls. GAPDH was used as a control. **(C)** Immunofluorescence assay indicated that ECA109 and KYSE150 cells transfected with siRNA PRMT5 significantly downregulated the PRMT5 expression compared with the controls. **(D)** CCK-8 assay in ECA109 and KYSE150 cells indicated that knocking down PRMT5 expression could inhibit the proliferation rate compared with the controls. **(E)** EdU assay in ECA109 and KYSE150 cells indicated that knocking down PRMT5 expression could inhibit the proliferation rate compared with the controls. Bars indicate the mean ± SEM, *P < 0.05, **P < 0.01 vs. control siRNA; magnification ×200. PRMT5, protein arginine methyltransferase 5; CCK-8, Cell Counting Kit-8.

It showed that the PRMT5 expression in clinical ESCC tissues and ESCC cell lines was abnormal, and we next explored the effects of PRMT5 in ESCC cells by transfecting siRNA-PRMT5 in ECA109 and KYSE150 cells. Transfection with siRNA-PRMT5 markedly downregulated PRMT5 mRNA and protein expressions, compared with the control siRNA (Figures 2A–C).

The CCK-8 assay and EdU assay were used on ECA109 and KYSE150 cells in order to evaluate the impact of PRMT5 on ESCC cell proliferation. CCK-8 assay showed that knocking down PRMT5 obviously inhibited the cell proliferation by 80.9% and 85.2% at 72-h point in ECA109 and KYSE150 cells, compared with the control siRNA (Figure 2D). In addition, EdU assay was also revealed that the percentage of positive proliferative cells in siRNA-PRMT5 group was significantly weakened, which indicated that low expression of PRMT5 suppresses cell proliferation (Figure 2E).

### PRMT5 Inhibited Esophageal Squamous Cell Carcinoma Cell Apoptosis

**FIGURE 3:**
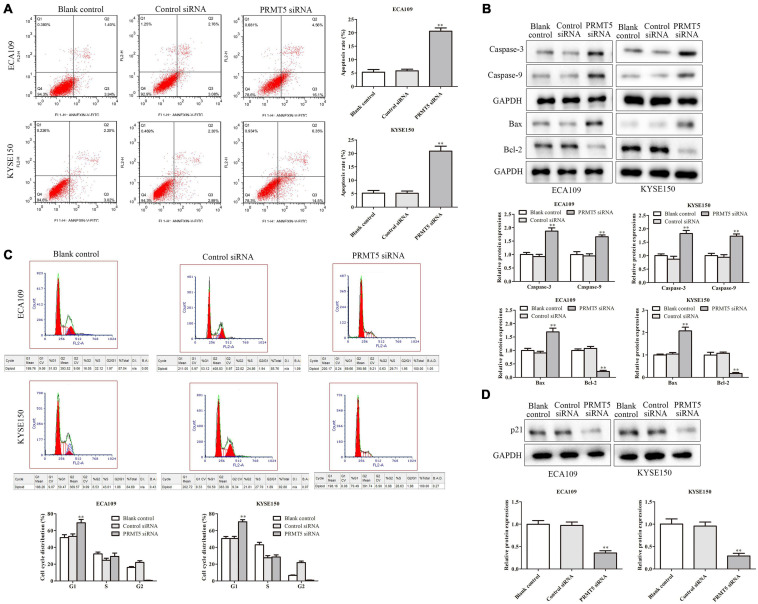
Knocking down PRMT5 promoted ESCC cell apoptosis and induced ESCC cell arrest in G1 phase. **(A)** The apoptosis rates of ECA109 and KYSE150 cells transfected with siRNA PRMT5 were detected by flow cytometry. **(B)** The expression levels of caspase-3, caspase-9, Bax, and Bax-2 were analyzed in ECA109 and KYSE150 cells. **(C)** Knocking down PRMT5 in ECA109 and KYSE150 cells significantly increased the G1 phase of cell cycle compared with the controls. **(D)** The expressions of p21 were analyzed in ECA109 and KYSE150 cells. GAPDH was used as a control. Bars indicate the mean ± SEM, *P < 0.05, **P < 0.01 vs. control siRNA. ESCC, esophageal squamous cell carcinoma; PRMT5, protein arginine methyltransferase 5.

To further explore the role of PRMT5 in ESCC cell apoptosis, flow cytometry analysis was applied. The cells in the upper-right (UR, Q2) and lower-right (LR, Q3) quadrants of the fluorescence-activated cell sorting (FACS) histogram represent apoptotic cells after culture for 48 h. As shown in Figure 3A, after being transfected with siRNA-PRMT5, the apoptosis rates of ECA109 and KYSE150 cells were 20% and 21%, respectively, which were significantly higher than those of the controls, indicating that down-expression of PRMT5 could induce cell apoptosis. In addition, compared with the controls, downregulating PRMT5 dramatically upregulated the expression levels of Bax, caspase-3, and caspase-9, while Bcl-2 level was downregulated in ECA109 and KYSE150 cells (Figure 3B). Taken together, PRMT5 could inhibit the cell apoptosis of ESCC.

### Knocking Down PRMT5 Induced Esophageal Squamous Cell Carcinoma Cell Arrest in G1 Phase

Furthermore, flow cytometry was also used to test the activity of PRMT5 on cell cycle in ESCC cells. After being transfected with siRNA-PRMT5, the rates of G1 in ECA109 and KYSE150 cells were 70% and 72%, respectively, which were significantly higher than the control siRNA, indicating that knocking down PRMT5 could induce cell arrest in G1 phase in both ECA109 and KYSE150 cells (Figure 3C). Besides, knocking down PRMT5 could downregulate the level of p21, compared with the blank and control-siRNA group (Figure 3D).

### PRMT5 Promoted Esophageal Squamous Cell Carcinoma Cell Migration and Invasion

**FIGURE 4:**
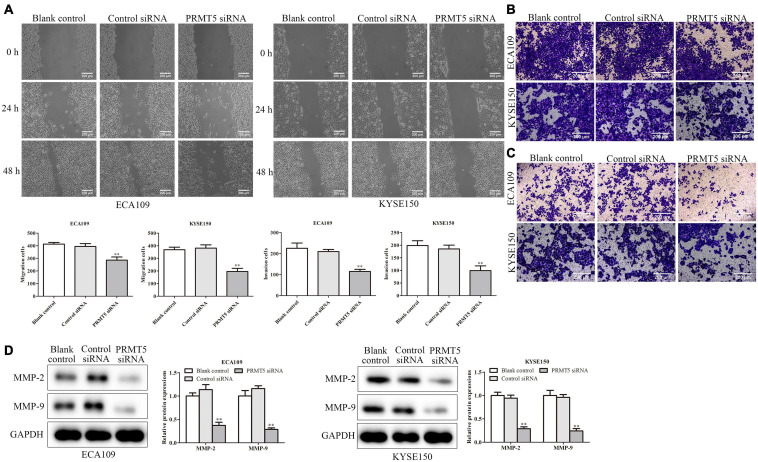
PRMT5 promoted ESCC cell migration and invasion. **(A)** Wound-healing assay indicated that knocking down PRMT5 expression effectively inhibited cell mobility. The photographs were taken at the magnification of ×40. **(B,C)** Transwell migration and invasion assay showed that knocking down PRMT5 expression suppressed the migration and invasion abilities of ECA109 and KYSE150 cells. The photographs were taken at the magnification of ×200. **(D)** The expression levels of MMP-2 and MMP-9 were analyzed in ECA109 and KYSE150 cells. GAPDH was used as a control. Bars indicate the mean ± SEM, *P < 0.05, **P < 0.01 vs. control siRNA. PRMT5, protein arginine methyltransferase 5; ESCC, esophageal squamous cell carcinoma.

The wound-healing and transwell assays were used to investigate the effect of PRMT5 in ESCC cell migration. It demonstrated that downregulation of PRMT5 inhibited the migration of ECA109 and KYSE150 cells (Figures 4A,B). Furthermore, invasion assay was performed to measure the effect of PRMT5 in ESCC cell invasion. As shown in Figure 4C, the number of invasion cells was reduced after transfection with siRNA-PRMT5 in both ECA109 and KYSE150 cells. To further determine the effect of PRMT5 in ESCC cell invasion, western blotting was adapted to determine the MMP-2 and MMP-9 levels. With downregulation of PRMT5, the levels of MMP-2 and MMP-9 were dramatically downregulated in ECA109 and KYSE150 cells, compared with the blank and control-siRNA group (Figure 4D). Overall, these findings indicated that PRMT5 promoted the cell migration and invasion in ESCC.

### PRMT5 Activated LKB1/AMPK/mTOR Signaling Pathway

**FIGURE 5:**
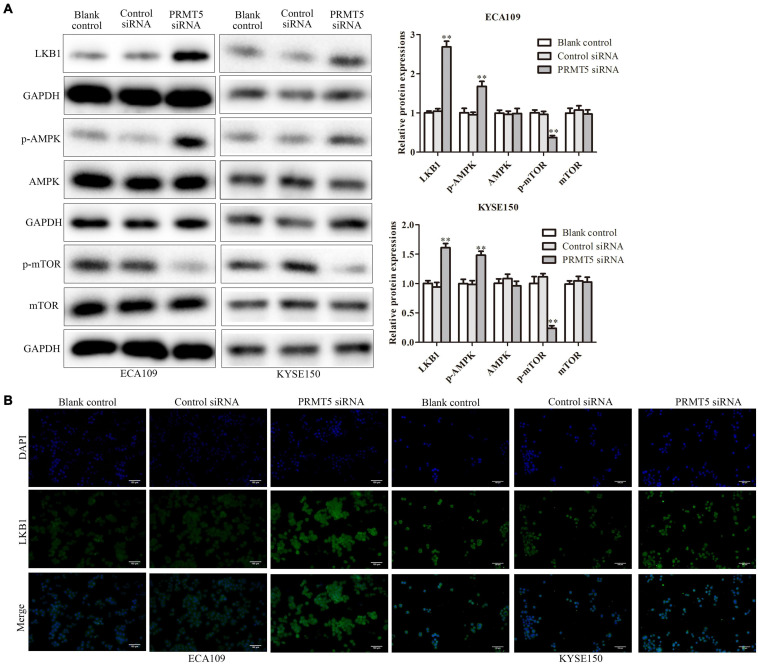
PRMT5 activated LKB1/AMPK/mTOR signaling pathway. **(A)** The expression levels of LKB1, AMPK, p-AMPK, mTOR, and p-mTOR were analyzed in ECA109 and KYSE150 cell lines. GAPDH was used as a control. **(B)** Immunofluorescence was used to determine the expression level of LKB1 in ECA109 and KYSE150 cell lines. Bars indicate the mean ± SEM, *P < 0.05, **P < 0.01 vs. control siRNA; magnification ×200. PRMT5, protein arginine methyltransferase 5; LKB1, liver kinase B1; AMPK, adenosine monophosphate kinase; mTOR, mammalian target of rapamycin.

To further investigate the mechanism of PRMT5 in ESCC, the expressions of LKB1, AMPK, and mTOR were examined. It showed that the knocking down PRMT5 significantly promoted the expression of LKB1 and the p-AMPK expressions in both ECA109 and KYSE150 cells, whereas the p-mTOR expressions were weakened after transfection with PRMT5 siRNA, compared with the controls (Figure 5A). Furthermore, the result of immunofluorescence indicated that the expression of LKB1 could be promoted by downregulating PRMT5 (Figure 5B). It suggested that PRMT5 could inhibit LKB1/AMPK/mTOR signaling pathway.

### LKB1 Inhibited Esophageal Squamous Cell Carcinoma Cell Proliferation, Migration, and Invasion and Promoted Esophageal Squamous Cell Carcinoma Cell Apoptosis

**FIGURE 6:**
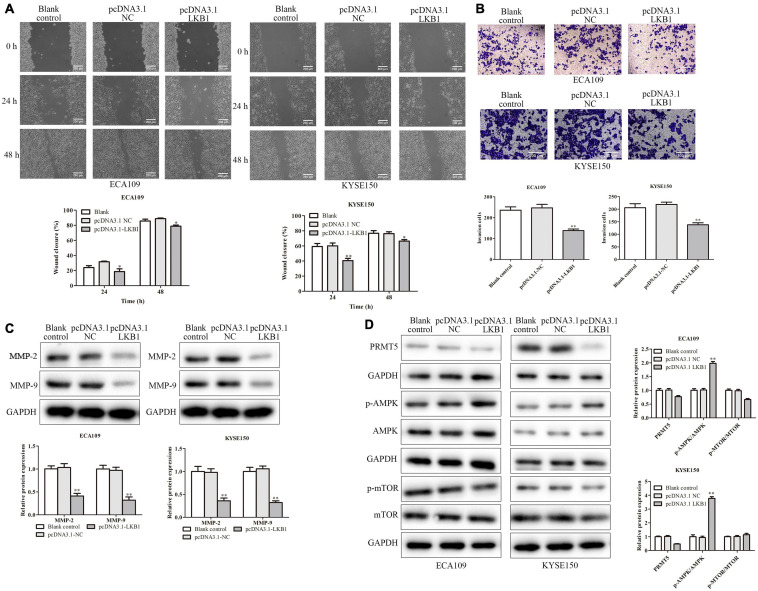
LKB1 inhibited cell migration and invasion. **(A)** Wound-healing assay indicated that overexpression of LKB1 effectively inhibited cell mobility. The photographs were taken at the magnification of ×40. **(B)** Transwell invasion assay showed that overexpression of LKB1 decreased cell invasion abilities. The photographs were taken at the magnification of ×200. **(D)** The expressions of PRMT5, AMPK, p-AMPK, mTOR, and p-mTOR were analyzed in ECA109 and KYSE150 cells. **(C)** The expression levels of MMP-2 and MMP-9 were analyzed in ECA109 and KYSE150 cells. GAPDH was used as a control. Bars indicate the mean ± SEM, *P < 0.05, **P < 0.01 vs. pcDNA3.0 NC. LKB1, liver kinase B1; PRMT5, protein arginine methyltransferase 5; AMPK, adenosine monophosphate kinase; mTOR, mammalian target of rapamycin.

To further explore the mechanism of LKB1 on ESCC, we performed an experiment by overexpression of LKB1 in ECA109 and KYSE150 cells. The transfection efficiency of LKB1 overexpression vector was identified by RT-PCR, and results have shown that the PRMT5 expressions were dramatically increased (Figures 6A,B). CCK-8 assay demonstrated that overexpression of LKB1 could suppress cell proliferation (Figure 6C). In addition, the result of flow cytometry analysis showed that overexpression of LKB1 could enhance the cell apoptosis rates in both ECA109 and KYSE150 cells, compared with the controls (Figure 6D). Furthermore, compared with the control, overexpression of LKB1 markedly upregulated the expression levels of Bax, caspase-3, and caspase-9, while Bcl-2 expression level was downregulated in ECA109 and KYSE150 cells (Figure 6E). Taken together, overexpression of LKB1 could promote the ESCC cell apoptosis.

**FIGURE 7:**
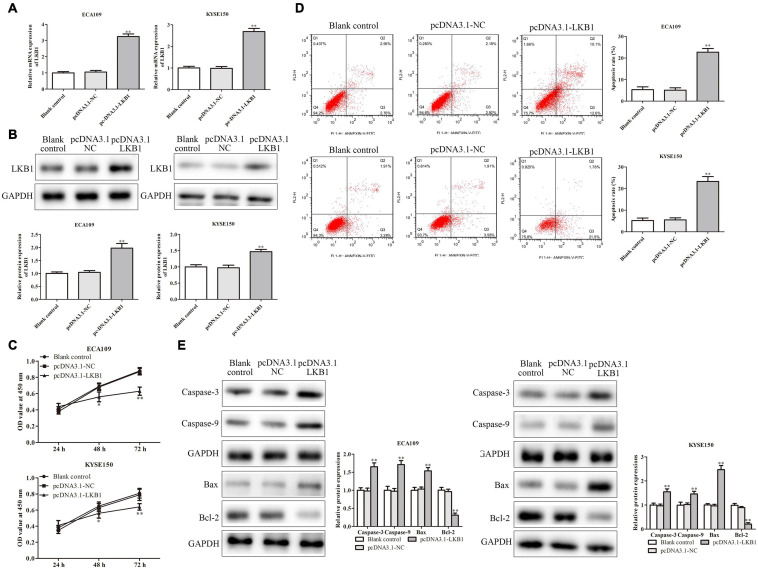
LKB1 inhibited ESCC cell proliferation and promoted ESCC cell apoptosis. **(A,B)** The expression of LKB1 in ECA109 and KYSE150 cells transfected with pcDNA-LKB1 was examined by RT-PCR and western blotting assays, respectively. **(C)** Cell proliferation rates were examined by CCK-8 assay in ECA109 and KYSE150 cells. **(D)** The apoptosis rates of ECA109 and KYSE150 cells transfected with pcDNA-LKB1 were detected by flow cytometry. **(E)** The expression levels of caspase-3, caspase-9, Bax, and Bax-2 were analyzed in ECA109 and KYSE150 cells. GAPDH was used as a control. Bars indicate the mean ± SEM, *P < 0.05, **P < 0.01 vs. control siRNA. LKB1, liver kinase B1; ESCC, esophageal squamous cell carcinoma; CCK-8, Cell Counting Kit-8.

Furthermore, the effect of LKB1 in ESCC cell migration and invasion was experimented on. And ECA109 and KYSE150 cells transfected with LKB1 overexpression vector could decrease the number of migrated cells and the invading cells (Figures 7A,B). Besides, with overexpression of LKB1, the MMP-2 and MMP-9 expressions were obviously downregulated in ECA109 and KYSE150 cells, compared with the blank and empty vector groups (Figure 7C). Last, we examined the expressions of key proteins from LKB1-related signaling. As a result, overexpression of LKB1 significantly upregulated the p-AMPK expression while downregulating the p-mTOR expressions in both ECA109 and KYSE150 cells, compared with the blank and empty vector groups (Figure 7D). Overall, it revealed that LKB1 could inhibit ESCC cell proliferation, migration, and invasion and induce cell apoptosis.

### PRMT5 Promoted the Tumor Growth *in vivo* via LKB1/AMPK/mTOR Signaling Pathway

**FIGURE 8:**
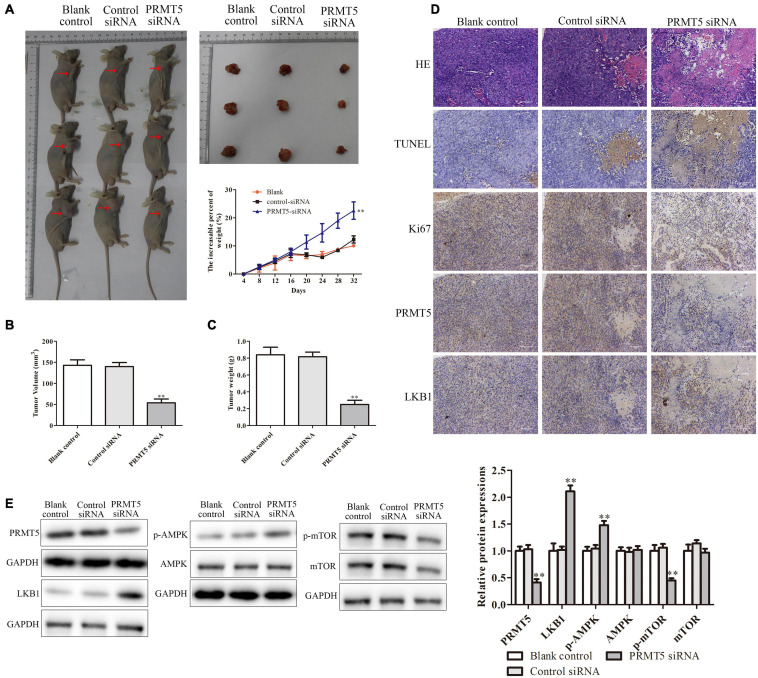
PRMT5 promoted the tumor growth *in vivo* via LKB1/AMPK/mTOR signaling pathway. The transfected KYSE150 cells were injected into the axilla of the nude mice. **(A)** Mice, tumors, and mouse weight are shown. **(B,C)** Tumor volume and weight were measured in different groups. **(D)** Immunohistochemistry indicated the expressions of Ki-67 and PRMT5 were lower in mice injected with ESCC cells transfected with siRNA PRMT5, while the expressions of TUNEL and LKB1 were higher. The photographs were taken at the magnification of ×200. **(E)** The expression levels of PRMT5, LKB1, AMPK, p-AMPK, mTOR, and p-mTOR were analyzed in ECA109 and KYSE150 cells. GAPDH was used as a control. Bars indicate the mean ± SEM, *P < 0.05, **P < 0.01 vs. control siRNA. PRMT5, protein arginine methyltransferase 5; LKB1, liver kinase B1; AMPK, adenosine monophosphate kinase; mTOR, mammalian target of rapamycin; ESCC, esophageal squamous cell carcinoma.

To further test and verify the effect of PRMT5 on the ESCC *in vivo*, KYSE150 cells transfected with blank, control siRNA, and PRMT5 siRNA were injected into the axilla of the male Balb/c nude mice. In the PRMT5 siRNA group, compared with the controls, tumor volume and tumor weight were significantly increased but have no significant difference in the weight of mice (Figures 8A–C). Furthermore, with the analysis of histological and IHC in tumor sections, the levels of TUNEL and Ki-67 were lower in the PRMT5 siRNA group than the control siRNA, which indicated that knocking down PRMT5 could inhibit the tumor growth and induce the tumor cell apoptosis *in vivo* (Figure 8D). Besides, it also showed that the expression of LKB1 was significantly upregulated along with the lower PRMT5 levels in the PRMT5 siRNA group than the blank and control siRNA groups (Figure 8D). Additionally, western blotting showed that knocking down PRMT5 could dramatically upregulate the expression of LKB1 and the p-AMPK in tumor sections, while downregulating the expression of p-mTOR (Figure 8E). These results suggested PRMT5 induced the tumor growth of KYSE150 cells *in vivo* via LKB1/AMPK/mTOR signaling pathway.

### PRMT5 Promoted Lung Metastasis *in vivo* via LKB1/AMPK/mTOR Signaling Pathway

**FIGURE 9:**
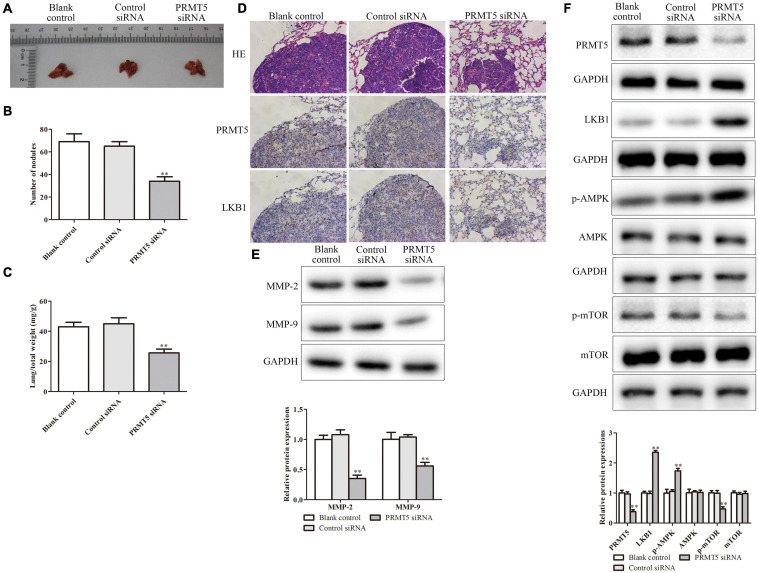
PRMT5 promoted lung metastasis *in vivo* via LKB1/AMPK/mTOR signaling pathway. The transfected KYSE150 cells were injected into the mice tail vein. **(A)** Lung tissues with metastatic foci were shown. **(B,C)** The number of nodules and lung/total weight were measured in different groups. **(D)** Immunohistochemistry indicated the expression of PRMT5 was lower, while LKB1 expression was higher in mice injected with KYSE150 cells transfected with siRNA PRMT5. The photographs were taken at the magnification of ×200. **(E)** The expression levels of MMP-2 and MMP-9 were detected by western blotting. **(F)** The expression levels of PRMT5, LKB1, AMPK, p-AMPK, mTOR, and p-mTOR were analyzed in ESCC cell lines. GAPDH was used as a control. Bars indicate the mean ± SEM, *P < 0.05, **P < 0.01 vs. control siRNA. PRMT5, protein arginine methyltransferase 5; LKB1, liver kinase B1; AMPK, adenosine monophosphate kinase; mTOR, mammalian target of rapamycin; ESCC, esophageal squamous cell carcinoma.

The lung metastasis model was performed to detect the effect of PRMT5 on ESCC metastasis *in vivo.* The lung metastatic foci and lung/total weight dramatically reduced in the PRMT5 siRNA group (Figures 9A–C). Furthermore, the expressions of PRMT5 and LKB1 in lung tissue dramatically downregulated in the PRMT5 siRNA group (Figure 9D). It showed that the levels of LKB1 and phosphorylation expressions of AMPK were obviously upregulated, while phosphorylation expressions of mTOR were obviously downregulated in the PRMT5 siRNA group (Figure 9E). Besides, knocking down PRMT5 obviously downregulated the MMP-2 and MMP-9 expression levels (Figure 9F). Taken together, these results provide direct evidence supporting our hypothesis that PRMT5 promotes lung metastasis in mice via the LKB1/AMPK/mTOR signaling pathway.

## Discussion

PRMT5-mediated H3R8 and H4R3 methylation inhibits transcription of many tumor suppressor factors, such as RB family genes, resulting in increased cell survival and proliferation (Pal et al., 2004; Wang Z. et al., 2018). In addition, PRMT5 directly methylates p53, thereby affecting the transcriptional activity of these essential cell fat modulators, promoting cell growth, and inhibiting apoptosis (Scoumanne et al., 2009; Gu et al., 2012). PRMT5 is overexpressed in many cancers including melanoma, lung, gastric, ovarian, and colorectal cancers. Herein, our data provided that the PRMT5 level was significantly upregulated in ESCC clinical tissues and ECA109 and KYSE150 cell lines.

Multiple lines of evidence have proven that PRMT5 is attributed to carcinogenic function and have recently received a lot of attention as a potential therapeutic target for cancer and exert their diverse biological functions. For example, PRMT5 could regulate human lung cancer cell growth via targeting Akt signaling (Zhang et al., 2018). Moreover, PRMT5 and FOXP1 expression profile in invasive breast cancer patients undergo neoadjuvant chemotherapy (Su et al., 2020). Tan et al. (2020) have found that high PRMT5 expression is associated with poor overall survival and tumor progression in bladder cancer. However, whether or not PRMT5 participates in the regulation of ESCC has not been well elucidated. Therefore, we further explored the potential function of PRMT5 by employment of knockdown experiments. The results demonstrated that knocking down PRMT5 obviously suppressed the cell proliferation, migration, invasion, and cell arrest in G1 phase of ESCC. At the molecular level, the expressions of the matrix metalloproteinases MMP-2 and MMP-9 were dramatically downregulated when PRMT5 was knocked down. In addition, downregulating PRMT5 promoted cell apoptosis in ESCC and upregulated the levels of Bax, caspase-3, and caspase-9, while Bcl-2 level was downregulated. Moreover, knocking down PRMT5 could weaken the tumor growth and lung metastasis *in vivo*.

Activation of adenosine monophosphate kinase (AMPK) is an enzyme that plays an important role in insulin signaling, systemic homeostasis, and glucose and fat metabolism (Towler and Hardie, 2007). The active form of AMPK, p-AMPK, downregulates the energy expenditure process and increases the production of adenosine triphosphate (Li et al., 2015). Epidemiological studies have found that a decrease in AMPK activity is accompanied by an increase in cancer mortality (Motoshima et al., 2006). AMPK mediated cell cycle regulation by upregulating the p53–p21 axis and the TSC2-mTOR pathway (Fan et al., 2018). For example, metformin inhibits ESCC carcinogenesis by inhibiting the AMPK/mTOR pathway, including activation of AMPK and inhibition of downstream molecules such as p-mTOR and cyclin D1 expression (Pelgrom et al., 2019). In this study, it was shown that PRMT5 could inhibit the p-AMPK expression as well as promote the expression of p-mTOR in ECA109 and KYSE150 cells, suggesting that PRMT5 could inhibit LKB1/AMPK/mTOR signaling pathway. It was further verified by animal experiments.

Liver kinase B1 is a tumor suppressor that encodes a serine–threonine kinase that directly phosphorylates and activates AMPK, a cell metabolism, and growing bioenergy sensors (Pelgrom et al., 2019). For example, by targeting LKB1, knocking down miR-744 may activate AMPK followed by inhibition of the mTOR signaling pathway (Zhang M. et al., 2019). Besides, the chemokine CXCL17 potentiates malignant invasion and inhibits autophagy via LKB1–AMPK pathway (Wang et al., 2019). In addition, LKB1 silencing reduced p-AMPK and increased phosphorylation of protein kinase B (p-AKT), thereby promoting tumor cell proliferation and enhancing migration and invasion of colorectal cancer (Chen et al., 2019). Herein, it was revealed that knocking down PRMT5 could increase the expression of LKB1 and the p-AMPK and could decrease the p-mTOR. Additionally, overexpression of LKB1 could reveal anti-tumor effects in ESCC cell lines by inhibiting ESCC cell proliferation, weakening migration and invasion, and accelerating cell apoptosis. Besides, upregulating the level of LKB1 could increase the expressions of Bax, caspase-3, and caspase-9 and could weaken the expressions of MMP-2, MMP-9, and Bax-2. Moreover, knocking down PRMT5 could weaken the tumor growth and lung metastasis *in vivo* while upregulating the LKB1 expression and the p-AMPK level and downregulating the p-mTOR expression.

Overall, PRMT5 may act as a tumor-inducing agent in ESCC by modulating LKB1/AMPK/mTOR pathway signaling. We will further make deeper and more detailed studies about regulation mechanism of PRMT5 on ESCC in the future work, including the inhibition LKB1/AMPK/mTOR pathway and whether PRMT5-siRNA has anti-tumor growth.

## Conclusion

1. PRMT5 expression level was dramatically upregulated in ESCC clinical tissues as well as ESCC cell lines.
2. Knocking down PRMT5 obviously suppressed cell migration, invasion, proliferation, and cell arrest in G1 phase and promoted cell apoptosis in ESCC cells.
3. Overexpression of LKB1 could reveal anti-tumor effects in ESCC cell lines by inhibiting ESCC cell, migration, invasion, and proliferation and accelerating cell apoptosis.
4. PRMT5 may act as a tumor-inducing agent in ESCC by modulating LKB1/AMPK/mTOR pathway signaling.

## Data Availability Statement

The original contributions presented in the study are included in the article/supplementary material, further inquiries can be directed to the corresponding author.

## Ethics Statement

The animal study was reviewed and approved by the Animal Ethics Committee of Heze Municipal Hospital. Written informed consent was obtained from the individual(s) for the publication of any potentially identifiable images or data included in this article.

## Author Contributions

JH conceived and designed the study. YC and HL performed the literature search and data extraction. LZ and CZ drafted the manuscript. All authors read and approved the final manuscript.

## Conflict of Interest

The authors declare that the research was conducted in the absence of any commercial or financial relationships that could be construed as a potential conflict of interest.

## Footnotes

1 http://gepia.cancer-pku.cn/

## Abbreviations

AMPK: adenosine monophosphate kinase
ANOVA: analysis of variance
DAB: diaminobenzidine
EdU: 5-ethynyl-2 ′ -deoxyuridine
ESCC: esophageal squamous cell carcinoma
FBS: fetal bovine serum
H&E: hematoxylin/eosin
LKB1: liver kinase B1
NAA40: *N*-alpha -acetyltransferase 40
OD: optical density
PI: propidium iodide
PRMT5: protein arginine methyltransferase 5
RT-PCR: real-time PCR
TdT: terminal deoxynucleotidyl transferase
TNBC: triple-negative breast cancer.
